# Non-psychologist practitioners’ views of cognitive assessment reports: An evaluation of a learning disability child and adolescent mental health service

**DOI:** 10.1177/17446295241228728

**Published:** 2024-01-27

**Authors:** Rebecca Hinch, Freya Hill, Martha Laxton-Kane

**Affiliations:** 152609 The University of Sheffield Faculty of Social Sciences, UK; Salford Care Organisation, Northern Care Alliance NHS Foundation Trust, UK; 2395 Chesterfield Royal Hospital NHS Foundation Trust, UK

**Keywords:** service evaluation, learning disability, mental health, qualitative, clinical psychology

## Abstract

This report details a service evaluation for a learning disability child and adolescent mental health service. The project aimed to explore nonpsychologist healthcare professionals’ views of the accessibility and utility of cognitive assessment reports produced in the service. Semistructured interviews were conducted with eight healthcare professionals. Thematic analysis identified three themes: value of reports, readability, and acknowledging multiple audiences, each with supplementary subthemes. The following were recommended: shorter reports; simpler language; examples and recommendations pertinent and applicable to individual clients (supported by the case holder if the assessor is unfamiliar with the client); visual information to support written text; assessor to provide verbal feedback in addition to the written report; main report should contain information most pertinent to families and professionals: the clients’ level of functioning/abilities and recommended interventions, whilst the appendix should contain supplementary information such as scoring and performance on individual subtests.

## Introduction

Undertaking psychological assessments to inform formulation and intervention is common practice in mental health services - learning disability services are no different. Psychological assessment reports can provide a fundamental basis for wider care teams regarding what recommendations to make in terms of service input and intervention (Hass and Carriere, 2014). According to Brown-Chidsey and Steege (2005, p.267), the usefulness of an assessment commonly relies on the findings of a report being shared with individuals in a position to employ solutions. Traditionally, research of psychological assessments has focused on assessing the reliability and validity of such measures, however, clinical utility research aims to evaluate the functionality and value of these measures in clinical practice (Brenner, 2003).

This evaluation focuses on a learning disability Child and Adolescent Mental Health Service (CAMHS) commissioned to meet the needs of children with a moderate to severe learning disability. The service consists of a multidisciplinary team of clinical psychologists, assistant psychologists, specialist learning disability nurses, specialist support workers, speech and language therapists, autism practitioners, psychiatrists, and trainee clinical psychologists.

As part of their role, the psychological professionals (psychologists, assistant psychologists, and trainee clinical psychologists) in the service conduct cognitive assessments, such as the Wechsler Intelligence Scale for Children Fifth Edition (WISC-V; Wechsler, 2014), and produce reports detailing the findings of these assessments. These reports are disseminated to others involved in the client’s care, such as parents/caregivers, general practitioners, social workers, and school nurses. Other members of the learning disability CAMHS service who are involved in the young person’s care, and actively deliver interventions to support clients, are also provided with the findings of these reports. Anecdotal reports from healthcare professionals within this service have suggested that cognitive assessment reports are difficult to understand, perhaps indicating that the accessibility and/or utility of these reports is limited.

In terms of writing psychological reports, the health and care professions council (HCPC; 2018) provides standards on how to communicate with service users, carers, and other professionals. Within this, the HCPC (2018) recommends that professionals must consider the needs and desires of service users and carers and provide information they want, or need, in a way that they can understand. Professionals are also expected to meet service users’ and carers’ language and communication needs where possible. Furthermore, the guidance states that professionals should work in partnership with colleagues, sharing knowledge, experience, and skills for the benefit of service users and carers, where appropriate. Additionally, it is recommended that relevant information should be shared with colleagues involved in the care and treatment of, or provider of other services to, the service user where appropriate.

More specifically, Pearson (2015) has created a “WISC-V Interpretive Considerations for Sample Report” document, which includes guidance for professionals on how to write WISC-V (Wechsler, 2014) reports for clinicians and for parents. Within these sample reports, the reader is expected to have the ability to learn and understand highly scientific and statistical concepts such as composite and primary index scores, verbal comprehension index, fluid reasoning index, and working memory index, and the different descriptive ranges of scores.

Psychological assessment reports are frequently criticised for being difficult to read for healthcare professionals (Groth-Marnat, 2009), teachers (Pelco et al., 2009), and parents (Wiener and Kohler, 1986). Barriers to understanding psychological reports, and the importance of clearly written reports, has been stressed for over 50 years (Martin, 1972), with some suggesting that a report’s efficacy is determined by the extent to which it is consumer-focused (Smith-Harvey, 2006) and produces meaningful change in terms of understanding and supporting clients (Ownby and Wallbrown, 1983).

Groth-Marnat and Horvath (2006) provide a review of some primary challenges impacting effective psychological report writing. These include: use of complicated language/jargon, report length, interpretation of included test scores, and generic interpretation of test scores that fail to link to the specific client. To gain a better understanding of the challenges of report writing, Smith-Harvey (2006) surveyed recently qualified clinical psychologists. In line with Groth-Marnat and Horvath’s (2006) review, participants highlighted that reports frequently contain jargon that may limit their accessibility. However, participants suggested that utilising simpler language in their reports could reduce the perceived credibility of the report. Smith-Harvey (2006) suggests that the study’s findings perhaps allude to difficulties in producing reports that are designed for, and utilised by, multiple individuals including other healthcare professionals, teachers, and parents, who may have varying levels of experience and knowledge which could aid or limit their understanding.

To explore the anecdotal reports emerging in this learning disability CAMHS team, the current evaluation aimed to identify the views of non-psychologist healthcare professionals with regards to the accessibility and utility of cognitive assessment reports. Namely the evaluation intended to ascertain what is helpful and unhelpful about the reports, with a view to improve these.

## Methodology

### Design

The current study utilised a qualitative, semi-structured interviews design to explore practitioners’ views of the cognitive assessment reports.

### Sample

The study utilised convenience sampling methods. Eligible participants had to work directly with clients on their caseload, use cognitive assessment reports in their work and have had no training or experience in administering cognitive assessments themselves. Eight members of the team met these criteria and were contacted via email. All eight potentially eligible team members consented to participate. The sample size was deemed sufficient in line with guidance from Malterud, Siersma, and Guassora (2016) as the aim of the study was narrow and the participant sample held characteristics highly specific for the study aim. All individuals who were eligible to participate did so. Participant characteristics are in Table 1.

**Table 1.** Sample Characteristics.

Job Role	Duration in Current Role (years)	Number of Cognitive Reports Viewed
Specialist Learning Disability Support	13	30+
Worker		
Specialist Learning Disability Support	14	30+
Worker		
Speech and Language Therapist	1.5	30+
Learning Disability Nurse Specialist	12	30+
Mental Health Nurse	7	30+
Lead Learning Disability Nurse	14	30+
Consultant Psychiatrist	1.5	30+
Consultant Psychiatrist and CAMHS	15	30+
Clinical Director		

### Ethical considerations

NHS ethical approval was not required as this work is classified as evaluative rather than research. This is consistent with the Health Research Authority (2017) guidelines for defining research. The project was approved by the Clinical Leadership Team and a commissioning form was approved by the University’s Research Support Officer. Participants were provided with an information sheet and completed consent forms prior to engaging in the evaluation.

### Interview schedule

The interview schedule was created by the primary author in collaboration with the commissioner and Assistant Psychologist. Topics were chosen in line with the research aim, and care was taken to avoid leading and closed questions. The interviews were conducted by either the primary author or Assistant Psychologist. All interviews took place over Microsoft Teams and recorded and transcribed verbatim by this software. Transcripts were reviewed by the authors against the recordings to ensure transcription accuracy. Transcript files were password protected and saved on a secure server. All video recordings were deleted after transcripts were reviewed for accuracy. Interviews lasted between seven and 25 minutes.

Prior to their interviews, participants were provided with an anonymised version of a cognitive assessment report produced recently. The example cognitive assessment report consisted of over five pages of text, with no images. The report consisted of the client’s reason for referral, their presentation during the assessment, their general cognitive ability (with an explanation of the WISC-V and the five cognitive domains that this measures), tables of scaled scores for each subtest and standard scores, percentile rank, and a qualitative description of each domain. Additionally, the reports detailed recommendations to promote skills under each cognitive domain, and information on how their difficulties might affect them in their everyday lives. The commissioner and Assistant Psychologist deemed this report as representative and typical of reports produced by the service as it utilised the existing report template created by the service. Participants were tasked to read this report prior to the interview to familiarise themselves with its contents and were asked during the interview to consider their perceptions of all cognitive assessment reports they had come across during their time in the service.

### Analysis

The current evaluation utilised an inductive approach whereby the data content drove coding and theme development. Interview data was analysed, and themes and subthemes were generated using Braun and Clarke’s (2006) six-phase framework for thematic analysis.

All transcripts were read through repeatedly by the primary author and Assistant Psychologist to enable data familiarisation. The transcripts were then divided between the primary author and Assistant Psychologist for coding. Text relevant to the research question was coded, and codes were developed and modified throughout the coding process. After initial coding of each transcript, the primary author and Assistant Psychologist compared codes and modified them accordingly. Discrepancies between codes were resolved through discussion, with further revisions made until agreement was reached, resulting in a total of 32 codes. Codes were then examined by both the primary author and Assistant Psychologist and organised to generate initial themes. Initial themes were reviewed and modified to ensure they were coherent and distinct from each other before they were defined. Any disagreements between authors were resolved through discussion. Had these not been resolvable through discussion, the project commissioner would have been consulted to aid resolutions.

### Reflexivity statement

The two authors who coded the transcripts acknowledge that being a part of the team in focus may have influenced their coding and theme development. The primary author was a temporary member of staff on placement in the team whilst completing a doctorate in clinical psychology. As a trainee clinical psychologist, the primary author has worked with children, young people, and adults with a learning disability, as well as having been trained in administering, interpreting, and constructing cognitive assessment reports, such as those that the study focuses on. At the time of the evaluation the secondary author had worked in this Learning Disability CAMHS for one year as an assistant psychologist and also has experience in administering, interpreting and constructing cognitive assessment reports. Steps were taken to remain objective, for example the data being analysed was anonymised prior to coding, and the authors utilised supervision to reflect on how their clinical experience may have influenced their assumptions or beliefs within this evaluation. Additionally, prior to commencing the evaluation, an open conversation was had between all the authors around how the evaluation’s findings might affect the team, and the importance of being open to change.

## Results

**Figure 1.**
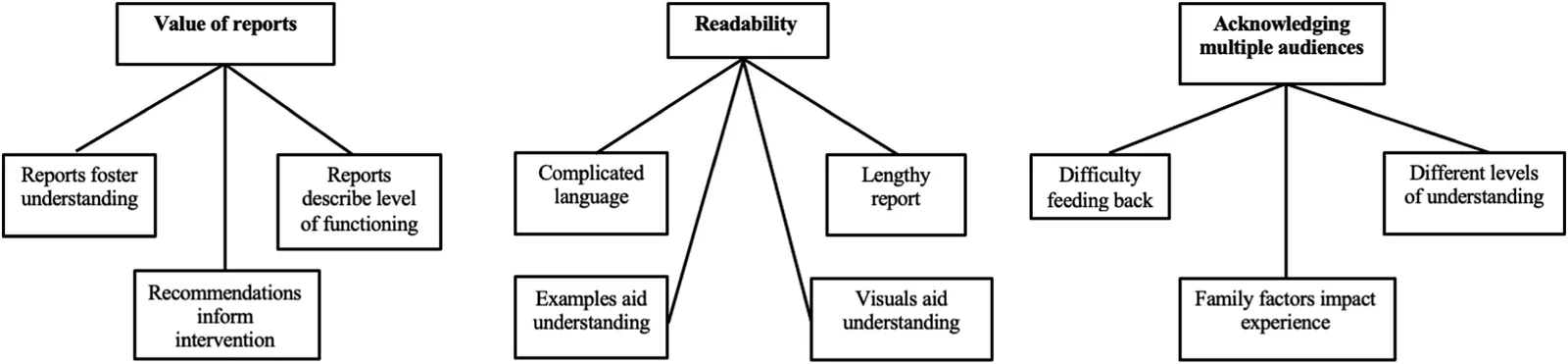
Themes and subthemes.

Thematic analysis identified three main themes with supplementary subthemes (Figure 1), described below with illustrative quotes. The themes relate to what participants wanted from reports, barriers to reading and understanding reports, and participants’ awareness of the varied levels of understanding amongst different parties who may receive reports.

### Value of reports

#### Reports foster understanding

Finding the reports to be helpful was commonly reported by participants, both for themselves in their professional capacity, and for parents and others involved in young persons’ care. Four professionals described finding them helpful in informing their work, and three described how informative they were for parents.

*“From a professional perspective, they’re very useful… and parents who I work with value them too”* Participant 2

*“I actually think that most children that come into our service should have one to be honest, because it gives everyone involved a much better understanding of the child and how the child learns and what they understand etcetera.”* Participant 6

Two participants described using the reports as part of their wider, systemic assessment, and one described utilising the recommendations section of the report to assist them in undertaking their work.

*“I mainly see them as part of an EHCP assessment or as part of a larger CAMHS assessment”* Participant 2

*“Having a report before I meet them to do my assessment helps as it informs a much wider, holistic understanding and formulation of the person. It’s part of the process… and the recommendations often highlight to me things I need to be aware of when I’m doing my assessment”* Participant 4

#### Recommendations inform intervention

All participants highlighted the importance and utility of the recommendations section, with six participants reporting that they utilise the recommendations to inform their work.

“*Interviewer: what sections of the report, if any, do you find useful?*

*“Participant: The recommendations. Because what I really need to know is, what do I need to do to adapt my practice to help the young person?”* Participant 8

*“I don’t look at the scores, mostly I just look at the recommendations and summary, because I need to know how best to work with them to help them reach their potential”* Participant 5

Three participants also stressed the importance of making the recommendations relevant to the individual, as they appeared to view this as more accessible and useful for others.

*“Making links with… everyday situations for young people makes it more accessible. So the more examples that can be used of the young person, like an actual example of what we know about that young person, helps parents to understand… because they can make that link to their actual child.”* Participant 1

*“Rather than [the report] just giving a number… it needs to provide an understanding and ideas of how to support that young persons’ particular cognitive profile”* Participant 4

#### Reports describe level of functioning

Three participants also expressed a need for reports to outline the young person’s level of functioning, as this appears to be helpful for themselves and other stakeholders to understand.

*“What’s not helpful is sometimes it’s not clear what [the young persons’] overall IQ score means, and whether or not it’s a definite definition of somebody’s level of disability, so whether or not [the report] shows that someone has a diagnosis of a moderate to severe learning disability”* Participant 3

*“I just want to know the result, like if someone’s got a mild, moderate, or severe level of learning disability and how this impacts the specific person”* Participant 5

### Readability

#### Lengthy report

Seven participants highlighted the lengthiness of reports to be a barrier for themselves and for families in terms of accessing the reports.

*“I just find it really time-consuming reading through one…it’s not that I would struggle normally, but there’s a lot to it, I had to read through it and read through it again to understand what [the report] is getting at… and if I’m struggling with that, I know families will be struggling with it”* Participant 8

*“[The reports] are fairly lengthy and I think that’s probably difficult for families as well…they can be really long”* Participant 6

Additionally, participants reported that, due to the length of the reports, it is unlikely that other professionals would have the time to read through them.

*“I would suspect that GPs or paediatricians would go straight to the bottom, to the summary, and maybe look at the overall scoring… but I wouldn’t imagine that they’ve got time to read all of this”* Participant 6

#### Complicated language

All participants reported difficulties with understanding language and terms used in the reports, which sometimes left them wondering how they could apply the report’s findings to the young person it related to.

*“It’s difficult…if it was another psychologist reading [the report] I imagine it makes complete sense. Whereas if it’s a family member who hasn’t got the experience of knowing what matrix reasoning is… you’re not going to understand”* Participant 5

*“A lot of the families just don’t have a concept of what a centile is. So even just throwing in some of those terms into a report actually makes it hard for parents to understand”* Participant 7

Two participants described needing to seek support from colleagues or having to search for terms on the internet to aid their understanding.

*“[The reports] have lots of words I don’t understand, I just can’t retain what those words mean, and I’m guessing parents wouldn’t… if we can just make it more user-friendly for reading, with less jargon in it, because… I often have to go to a colleague in psychology to help me understand them”* Participant 3

*“I think some terminology is not so helpful… ‘rote learning’ wasn’t a term I was familiar with, I had to Google it, even though I’ve read these reports before”* Participant 8

#### Visuals aid understanding

Two participants identified that reports could be enhanced and made more accessible if they included diagrams to support the text.

*“I’m a visual learner, all that writing is really difficult for me to absorb. Information presented in diagrams or charts, is good for me… like if there was some way to indicate the level of learning disability with a visual, that would be helpful”* Participant 8

*“If there was a more pictorial reference to where [the young person] is in terms of their ability range, it might mean more than giving them a score or a centile or something”* Participant 7

#### Examples aid understanding

Five participants suggested examples made reports more accessible and useful for themselves and for families who want to know how to apply the reports’ findings.

*“When it talks about ‘visual spatial, fluid reasoning’ etcetera, although it kind of makes sense to me, I could do with an example really*.*”* Participant 8

*“A lot of it’s jargon, and it can be difficult to know what it actually means, but if there’s a sentence to define what it means, and what it means for that person, then that’s helpful”* Participant 6

*“Examples that are about the young person can help parents to understand what [the report] means in terms of the young person’s functioning”* Participant 1

### Acknowledging Multiple Audiences

#### Differing levels of understanding

The cognitive assessment reports are shared with various parties involved with a young person’s care. Seven participants recognised that different people who would read the report would have differing levels of understanding.

*“Different professionals have different specialisms and different skills, so some would find it easier to interpret than others”* Participant 2

*“I think how likely it is for someone to understand depends on their skills and previous training experience”* Participant 4

Three participants suggested that different individuals may require different information from reports.

*“I think professionals need all this information, don’t we? We need it all to be on file for us. But I think we need to think about the messages that we give to families, because they don’t need all of this”* Participant 7

Three participants reported that their understanding of reports increased the more they came across them, which may suggest that families, who may not have seen such reports before, may struggle to access them.

*“It’s something that you need to be coming across routinely in your job, the more [the reports] do, the more they make sense.”* Participant 5

*“The more I’ve come across these reports, I obviously understand the language of them a lot more than what I did initially”* Participant 1

#### Family factors impact experience

Four participants described how factors relating to families may impact the experience of receiving reports. They noted that families of the young people being assessed may also have learning disabilities themselves, and participants suggested that reports are not currently written in a way that would be accessible to these individuals.

*“If you’ve got a parent who has a learning disability themselves, then that report is just not going to be accessible to them”* Participant 5

*“A lot of the parents have got their own learning difficulties, neurological problems, or genetic problems. When they get reports that run four to six sides, that would be overwhelming”* Participant 7

The anxiety that some family members may experience was also thought to be a potential barrier to understanding reports.

*“A lot of the families we might be dealing with are quite anxious, so they might not read through properly, they probably find a lot of it difficult”* Participant 8

Two participants also described the sensitive nature of the reports’ findings and how they might be experienced by families.

*“The phrasing is also difficult, because when it says, your child scored x, and out of 100 young people, 99 scored higher than your child… it’s difficult emotionally for parents to see that information written down in black and white.”* Participant 8

#### Difficulty feeding back

Seven participants commented on the process by which families and others receive reports. Participants suggested that families often do not have reports explained to them by the person who wrote them.

*“It’s emotionally difficult for parents, especially if they get [the report] sent out as a letter, and not given to them face-to-face to be spoken through and discussed”* Participant 8

Participants suggested that they often had to explain to others what the reports meant, even if they were unsure themselves.

*“I’ve gone through it with staff at school, because I think the teachers won’t understand it all, and I think if you can go through it with somebody, you can point out what they need to know for whatever role they play”* Participant 6

*“A lot of families show me the reports and ask me what it means, then I have to go all the way through it to find the key information, but I find it difficult to explain to families”* Participant 8

Six participants also suggested that an easy-read version of reports might increase accessibility.

*“It could be helpful to have different reports for different people… a more in-depth report for their file, but for parents and other professionals it just needs to say what you’ve done and really what is recommended to help the young person”* Participant 6

*“You could have one report like this, that goes into what was done… with all the jargon… and a separate one with the results and what it means for the child. So if they’re struggling with x then you can do y to help”* Participant 5

### Dissemination

After receiving written approval from the commissioner to disseminate the findings, feedback from the evaluation was presented to the learning disability CAMHS team in a team meeting.

## Discussion

This service evaluation aimed to explore non-psychologist practitioners’ views of the accessibility and utility of cognitive assessment reports produced by a learning disability CAMHS team.

Despite the reports being disseminated to healthcare professionals, families, and other professionals involved in a young person’s care, the findings of this evaluation suggest that all recipients experience some level of difficulty understanding the reports. The findings highlight issues with reports that may act as barriers for individuals to understand them: the use of complicated language and the length of reports. These findings are in line with previous research; for example, Horvath, Logan, and Walker (2002) found that the length of psychological reports varies widely, ranging from one to 54 pages. Longer reports have been described by parents and teachers as intimidating (Mastoras et al., 2011). Such research, in addition to the current findings, provide support for reports to be as concise as possible, perhaps by focusing on what is most important to the reader, as suggested by Donders (2016), or through the use of bullet points in place of blocks of text (Schneider et al., 2018). Furthermore, existing literature exploring both recipient and author perspectives has identified the use of complicated language as a potential barrier to accessing the reports (Brenner, 2003; Groth-Manat and Horvarth, 2006; Smith-Harvey, 2006) and can lead to misinterpretation of the findings (Rahill, 2018). Harvey (2006) offers multiple suggestions for making reports more readable, including: using shorter sentences, increasing subheadings use, and using less jargon. The current article proposes that, whilst psychological reports often address complex and novel topics, acronyms and the use of complex language should be avoided where possible in favour of more simple, shorter words.

Furthermore, participants acknowledged difficulties in producing reports for multiple audiences. Despite all recipients of reports wanting to understand and support the client, participant differences (such as levels of experience, knowledge, and familiarity with these types of reports) may affect the extent to which an individual can access the reports’ findings. Consequently, clinicians producing psychological reports should not assume that the report’s readers have prior understanding of the concepts covered in the reports. Participants also highlighted feedback mechanisms as an area of potential development, with many proposing families and professionals would benefit from discussing reports’ findings with the assessor. It is important to consider the wider adjustments required to facilitate such change, and whilst implementing this additional form of feedback may require more time and resources from healthcare professionals in the first instance, however, the benefits of this additional feedback mechanism should not be underestimated. Indeed, research suggests that clients who have contact with the assessor as well as written feedback from psychological assessments are considerably more satisfied with the process than those who only receive written feedback (Finn and Tonsager, 1992). Feedback may also increase the likelihood of recommendations being implemented (Salvagno and Teglasi, 1987).

Importantly, analysis revealed that participants felt the reports are valuable, both for themselves, and for others involved in the young person’s care, with participants utilising the reports’ findings, particularly the recommendations, to inform their own intervention work. Participants also highlighted the need for reports to include examples and recommendations that were relevant to clients in terms of abilities and interests. Indeed, Pope (1992) found that clients derived the greatest therapeutic benefit from reports which utilised examples relevant to the client’s life. Together, these findings suggest that, despite difficulties related to their accessibility, the reports do facilitate the ability to better understand and support clients which, according to Ownby and Wallbrown (1983), suggests that the reports are effective. Additionally, these findings suggest that it is important for clinicians to work alongside those involved in the young person’s care to develop practical recommendations that are both applicable and relevant (Groth-Marnat & Wright, 2016). Many services utilise templates to produce psychological reports for ease and consistency, however, this may be at the detriment of person-centred care. Wider organisational changes may be necessary in services to allow healthcare professionals the additional time required to produce reports that are more relevant and, therefore, more clinically useful, to the individual that the report is based upon.

On balance, the findings of this evaluation suggest that cognitive assessment reports produced in this learning disability CAMHS team are deemed valuable for supporting clients; however, there are multiple changes that can be implemented to increase accessibility of the reports for all recipients, these changes are outlined in greater detail the recommendations section below. It is imperative that mental health services that support individuals with a learning disability address the barriers to accessibility highlighted in the current article. Such services should explore the perspectives of the recipients of their psychological reports, including the perspectives of the young people on whom the reports are based and their families, and adapt them accordingly.

Additionally, this evaluation highlights wider considerations for psychologists and other healthcare professionals who produce reports and disseminate information to clients and others involved in their care. Perhaps, as suggested in Groth-Manat and Horvath’s (2006) review, healthcare professionals are under pressure to produce reports that utilise more complex language, due to a belief that using simpler language could reduce the report’s perceived credibility. The current research, in addition to Groth-Manat and Horvath’s (2006) review, may suggest that the aforementioned issues and barriers to understanding indicate a wider, systemic, organisational issue with the manner in which information is conveyed between healthcare professionals and their patients. Further research is needed to understand the mechanisms that lead to such reports being written in a manner that is not accessible to most individuals, which ultimately diminishes the value and negates the purpose of these reports.

### Critical review

This evaluation benefits from having two reviewers involved with the thematic analysis process. Utilising two individuals and having clear processes through which disagreements were resolved reduces the risk of bias during each stage of analysis.

A potential limitation of this evaluation is the smaller sample size, which may suggest that the findings should be interpreted with caution. However, many of the findings are in line with the wider evidence base and consequently provide support for the notion of implementing changes to reports to improve their accessibility and utility for all recipients.

This evaluation may also be limited in that participants were presented with one anonymised version of a cognitive assessment report. Whilst this report was deemed representative of a typical report produced in the service, participants may have been influenced to focus their comments on this specific report. Participants were asked during the interview to consider their perceptions of all cognitive assessment reports they had come across during their time in this particular service in an attempt to reduce bias.

A further limitation of this evaluation is the lack of involvement from individuals with a learning disability and other parties involved in caring for the young people who access the service. Although questions were asked about participants’ perceptions of the accessibility of reports for families and other professionals, future research would benefit from investigating this topic with those parties to explore their experiences of reports with greater accuracy. This may also aid the development of an easy read version of the reports so that this is accessible to the young person at the centre of the report, which may be imperative to developing their autonomy.

## Recommendations

The reports should be commensurate to all possible recipients. The following recommendations are intended to improve reports’ readability: the report should include shorter sentences, use less jargon and acronyms, minimise difficult words, increase the use of subheadings, and use everyday descriptions of clients’ behaviours. Examples relevant to the client and visual information should be included to support understanding of the written text. Visual information may also help to contextualise information that is novel to the reader, for example, diagrams may aid the readers’ understanding of how the clients’ scores compare to others of their age.

To make reports more accessible, they could be split into two sections: one containing information relevant to families such as the recommendations and what the assessment found in terms of the client’s abilities, and the other to include information that may support healthcare professionals, such as the client’s scores and performance on each subtest.

The reports’ recommendations were deemed most valuable by participants. Consequently, intervention recommendations should be clearly written, concrete and specific, whilst being relevant to the client (for example, through using examples in line with the client’s interests and abilities). If the assessor has only interacted with the client during the psychological assessment, the healthcare professional who has been working with the client and family could be consulted to ensure examples are relevant to the client.

In addition to the written report, it is recommended that families and other professionals receive feedback verbally from the assessor. It may be advantageous for the healthcare professional that is the case holder for the client to also be present for these discussions. This will allow report recipients the opportunity to ask clarifying questions and consider how the report’s recommendation could be implemented into everyday lives.

The service the current study focuses upon would benefit from conducting a further evaluation that explores the utility and accessibility of the reports from the perspectives of families and other professionals. An example report template that has taken into consideration this evaluation’s findings and recommendations is provided in the Supplementary Material.

Given the highlighted importance of psychological reports, and the potential for them to have a meaningful impact for children with a learning disability, it is imperative for learning disability services to review the accessibility and utility of their reports from the perspectives of the reports’ multiple recipients. Services may benefit from evaluating the effectiveness of their reports through qualitative methods (such as focus groups and semi-structured interviews) in the first instance, and make appropriate adaptations to their methodology based upon the aforementioned limitations of the current study. These evaluations should ensure to capture the perspectives of the different recipients of the report, and ensure that the voices of patients, caregivers, and communities who care for patients or utilise services, are heard. Patient and public involvement should be integrated throughout the research process, including the design stage. This can be achieved, for example, through the Partner Priority Programme (PPP) model which was developed by the National Health Service (NHS) and National Institute for Health Research Collaboration for Leadership in Applied Health Research and Care (NIHRCLAHRC) North West Coast and has been shown to be an effective model for embedding patient and public involvement within research which in turn reduces inequalities in healthcare (Saini et al., 2021). The current study’s findings may also have wider implications for other forms and processes of communication that healthcare professionals have with patients, and may highlight a need for healthcare providers to review such processes to ensure they are both accessible and useful for their intended recipients. Finally, at an organisational level, changes may be required to facilitate healthcare professionals to have the additional time and resources needed to create and feedback more person-centred reports.

## Conclusion

This article has explored the accessibility and utility of cognitive assessment reports produced in a learning disability child and adolescent mental health service from the perspective of the healthcare professionals whom utilise the reports to inform their interventions. The article outlines many limitations and barriers of existing reports, and highlights many changes that can be implemented to increase the accessibility and subsequent utility of these reports. These findings have important implications for the current service and for other services that work with this population. It is imperative that further research is conducted with the recipients of such reports to explore their perspectives, with an aim to produce psychological reports that are as accessible as possible.

## Supplemental Material

Supplemental Material - Non-psychologist practitioners’ views of cognitive assessment reports: An evaluation of a learning disability child and adolescent mental health service Supplemental Material for Non-psychologist practitioners’ views of cognitive assessment reports: An evaluation of a learning disability child and adolescent mental health service by Rebecca Hinch, Freya Hill,Martha Laxton-Kane in Journal of Intellectual Disabilities
