# Supplementary material for: Non-psychologist practitioners’ views of cognitive assessment reports: An evaluation of a learning disability child and adolescent mental health service
Source: J Intellect Disabil. 2024 Jan 27;30(3):414–28. doi: 10.1177/17446295241228728 (PMC13519066; doi:10.1177/17446295241228728)
Supplement: Supplementary file 1 [file sj-pdf-1-jld-10.1177_17446295241228728.pdf]

Supplementary material

Proposed Cognitive Assessment Report Template Based on the Evaluation's Findings

PRIVATE AND CONFIDENTIAL CLINICAL PSYCHOLOGY REPORT COGNITIVE ASSESSMENT

Re: DOB:
Address:

What we did & why

[Referrer] asked us to meet [Client] to look at the way they think about things. We did an assessment on [date] that took [x] minutes. The assessment tested [Client]'s thinking abilities and helped us to know what [Client] is good at, and what they might find difficult. It also shows us what level [Client] is working in comparison to other children their age.

In the Appendix there is more information about the assessment and [Client]'s scores. You may wish to share this with [Client]'s school or other healthcare professionals.

What we found

Delete as appropriate

[Client]'s scores suggest that they have a moderate-severe learning disability.
This scale shows where [Client]'s scores fit in relation to other children their age:

Score	69 and below	70-79	80-89	90-109	110-119	120-129	130+
Description	Extremely Low	Very Low	Low Average	Average	High Average	Very High	Extremely High

Full Scale IQ, Verbal Comprehension, Visual Spatial, Fluid Reasoning, Working Memory, Processing Speed

Because [Client]'s scores varied so much, we cannot give an overall score for their thinking ability (this is called a Full Scale IQ score). However, all [Client]'s scores were below average in comparison to other children their age.

What [Client] is good at

Edit as appropriate

- [Client] tried really hard and followed all of the instructions
- With a bit of encouragement, [Client] joined in with all the tasks
- [Client] was very pleasant to talk to

What [Client] finds difficult

- Sitting still without fidgeting
- Understanding what people mean when they talk (especially more abstract concepts)

- Being understood by others when they talk
- Understanding things quickly
- Remembering what someone has said, which can make it difficult to reply
- Understanding pictures and written information (like instructions on a whiteboard, maps, and signs)
- Learning new information

This may mean that [Client] finds conversations, social situation, and schoolwork more difficult. This may lead to [Client] experiencing low self-confidence, frustration and/or anxiety in these situations.

Recommendations

Choose recommendations that are relevant to the client, and edit the examples to fit the individual

Below are some strategies that can help to develop different areas of [Client]'s thinking abilities. You can use the boxes to note down which strategies you're going to try first.

Verbal Comprehension: to help [Client] develop her use and understanding of language. This includes 'receptive comprehension' (the ability to understand spoken or written language), 'expressive comprehension' (the ability to use words to express ourselves), and 'augmentative and alternative communication' (the ability to communicate using methods other than speaking, e.g. gestures, facial expressions, and communication aids):

- ☐ **Repeat instructions, using the same words, to help them to understand tasks**
  Example: "[Client], shoes on", "shoes on"
- ☐ **When giving instructions, break these down into simple steps (try not to give multi-step instructions)**
  Example: "[Client] put your coat on" then, once complete, "put your shoes on"
- ☐ **Use short easy words to help them understand what you're saying/asking**
  Example: "[Client], time for bed"
- ☐ **Help them to learn new words**
  Example: read books together about things that interest them, such as …

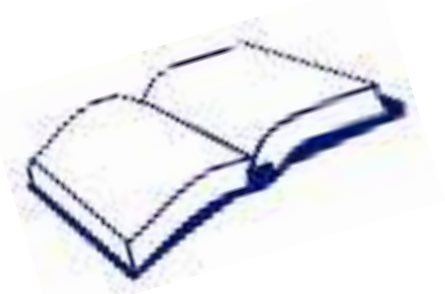

- ☐ **Help them to learn how to act in social situations**
  Example: social stories (like a comic strip) can show how to make friends and act in different situations
- ☐ **Teach them how to ask for help**
  Example: If [Client] can't say what she needs, give [Client] different flashcards with different actions or feelings on them to show you when she needs or feels something
- ☐ **Try to use questions that have fewer response options**
  Example: "reading or sensory room?", "yes or no?"

Visual Spatial: to help [Client] use and understand pictures and written information

- [ ] **Use pictures and words together**
  Example: point to a picture of school and say "school now"
- [ ] **Show [Client] what to do when you want them to complete a task**
  Example: if [Client] needs to do something, show her how to do it first, then say "now you do…"

Fluid Reasoning: to help [Client] problem solve, and apply their knowledge to know what to do in new situations and environments

- [ ] **Talk to [Client] about new or unexpected situations**
  Example: "Teacher A is not in school today. Teacher B will be teaching you today."
- [ ] **Try not to assume that [Client] knows how to do a task, even if she has done the same task somewhere else, or done something similar**
  Example: [Client] may know how to do something at home, but struggle with the same thing at school or in public
- [ ] **Use pictures and words together**
  Example: point to a picture of a bus and say "bus now"

Working Memory: to help [Client] remember visual and auditory information and to use this information in a task

- [ ] **Tell them what you want them to do, not what not to do**
  Example: "[Client], turn off your computer", not "[Client], stop playing on your computer"
- [ ] **Help [Client] make a daily planner or schedule, and remind them to look at it**
  Example: Monday morning might have pictures and words of [Client] waking up, getting dressed, eating breakfast, brushing teeth, and brushing hair
- [ ] **Put routines in place to help [Client] remember what to do**
  Example: after school, [Client] takes off her shoes, changes clothes, watches television, then has dinner
- [ ] **Use pictures to help [Client] remember what has been said**
  Example: say "we are going on the bus" and show a picture of a bus.
- [ ] **Keep language simple, so [Client] has less information to hold in mind. Break down big tasks/instructions into smaller ones and give [Client] one instruction at a time.**
  Example: "work finished, hand massage now"
- [ ] **Show [Client] how to relax through exercising, stretching, and taking deep breaths. Remembering things can be more difficult if someone is anxious or stressed.**

Processing Speed: to help [Client] process information

- [ ] **Give [Client] time to complete tasks**
- [ ] **Try to remove distractions around [Client] when asking them to do things**

Example: provide a quiet room when [Client] needs to concentrate

☐ **Give information in small chunks**

Example: say “[Client] school time. Put your shoes on”, wait until they have done this, then say “good job. Now put your coat on”

☐ **Give [Client] more time to respond in conversations. Pause between sentences to give [Client] more time to understand what was said**

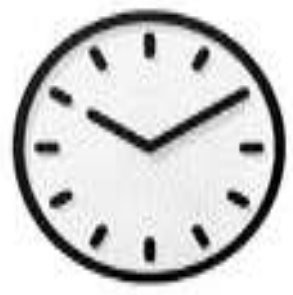

☐ **It is important for [Client] to know that it is ok to take more time to complete a task accurately. It is better for them to complete something slowly and correctly, than it is to rush through and make mistakes.**

Example: acknowledge [Client]’s ability to take their time and perform to the best of her ability. This may increase her self-esteem and motivation.

If you have any questions about anything in this report, or the Appendix, please contact LD CAMHS on: [telephone number]

Yours sincerely,
Name
Role

Appendix

The Weschler’s Intelligence Scale for Children, 5th Edition (WISC-V) was used to understand [Client]’s cognitive abilities. The WISC-V is made up of ten different tests that measure five areas of cognition. These areas, [Client]’s performance on the test, and their scores will be discussed here.

Verbal Comprehension

Definition: The ability to verbally express ourselves in a clear and efficient way.

For this ability, [Client] scored within the ***X*** range when compared to other people of her age.

What the tests involved: The Similarities subtest asked [Client] to explain how two words were alike. ***Describe performance.***

The Vocabulary subtest asked [Client] to explain the meaning of different words. ***Describe performance.***

What this means: ***Describe impact including strengths and weaknesses.***

Visual Spatial

Definition: the ability to understand and use visual information and details.

For this ability, [Client] scored within the **extremely low** range when compared to other people of their age.

What the tests involved: The Block Design task involves re-creating patterns out of coloured blocks. ***Describe performance.***

The Visual Puzzle subtest required [Client] to choose three puzzle pieces that could be put together to form a large picture. ***Describe performance.***

What this means: ***Describe impact including strengths and weaknesses.***

<u>Fluid Reasoning</u>

Definition: The ability to solve problems using logic and reasoning, and to apply knowledge to new or unfamiliar situations.

For this ability, [Client] scored within the ***X*** range when compared to other people of their age.

What the tests involved: The Matrix Reasoning task involves choosing a shape that would fit in a space and follow the pattern of the other shapes before it. ***Describe performance.***

The Figure Weights task involves choosing a shape to fit on a scale to balance the weight of a shape already present on one side of the scale. ***Describe performance.***

What this means: ***Describe impact including strengths and weaknesses.***

<u>Working Memory</u>

Definition: The ability to remember visual and auditory information in the short-term and to use this information in a task.

For this ability, [Client] scored within the ***X*** range when compared to other people of their age.

What the tests involved: The Digit Span task involves the examiner verbally presenting a sequence of number that increases in length. The first task requires respondents to repeat the number sequence in the same order, the second requires respondents to repeat the number sequence backwards, and the final task requires respondents to order the number sequence from lowest to highest. ***Describe performance.***

The Picture Span task involves pictures being presented to the respondent for 3-5 seconds, then the respondent is shown a number of pictures and asked to identify what picture was originally presented. As this task progresses, the young person has to remember multiple pictures and identify what order they were shown in. ***Describe performance.***

What this means: ***Describe impact including strengths and weaknesses.***

Processing Speed
Definition: How quickly you can process information.

For this ability, [Client] scored within the ***X*** range when compared to other people of their age.

What the tests involved: The Symbol Search task involves the young person having to identify whether a symbol is present or not in a line of different symbols. ***Describe performance.***

The Coding task involves the young person drawing a special mark that corresponds to a number. ***Describe performance.***

What this means: ***Describe impact including strengths and weaknesses.***

Assessment Observations

Brief paragraph on engagement: *For example:*

- *Did the child complete the assessment alone?*
- *Did the child complete all of the tasks presented to them? Were any strategies needed to promote engagement?*
- *Did the child find any tasks difficult or have a preference for certain tasks?*
- *Did the assessment need adapting in any way?*
- *Was the child anxious or self-conscious?*
- *How did they present? E.g. quiet, energetic, shy, talkative, polite, fidgety.*

Scores

Table 1: Summary of [Client]'s scores on the WISC-V

Scale	Standard Score*	Percentile rank**	Qualitative Description
**Verbal Comprehension**			
**Visual Spatial**			
**Fluid Reasoning**			
**Working Memory**			
**Processing Speed**			
**Full Scale IQ**			

*Standard scores: Scores which compare [Client]'s performance to a group of her peers of a similar age.
**Percentile ranks: Scores that indicate the number of children in a sample of one hundred who are 15 years and 7 months of age who would achieve the same or below [Client]'s score. For example, if [Client] scored on the 1st percentile, this would mean that 1% of a sample of same-aged peers scored the same or below, whilst 99% scored above.

Table 2: Summary of scaled scores on subtests of the WISC-V.

**Verbal Comprehension**	**Visual Spatial**	**Fluid Reasoning**	**Working Memory**	**Processing Speed**
Similarities	Block Design	Matrix Reasoning	Digit Span	Coding
Vocabulary	Visual Puzzles	Figure Weights	Picture Span	Symbol Search

* Scaled scores range from 1 to 19 with 8-11 being an average level of performance
